# Pedagogy Does Not Necessarily Constrain Exploration: Investigating Preschoolers’ Information Search During Instructed Exploration

**DOI:** 10.1111/desc.70004

**Published:** 2025-03-10

**Authors:** Rebeka Anna Zsoldos, Ildikó Király

**Affiliations:** ^1^ Doctoral School of Psychology ELTE Eötvös Loránd University Budapest Hungary; ^2^ Institute of Psychology ELTE Eötvös Loránd University Budapest Hungary; ^3^ MTA‐ELTE Social Minds Research Group, Institute of Psychology, ELTE Eötvös Loránd University Budapest Hungary

**Keywords:** exploration, guided play, learning, pedagogy

## Abstract

Pedagogy is seen as a “double‐edged sword”: it efficiently conveys information but may constrain the exploration of the causal structure of objects, suggesting that pedagogy and exploration are mutually exclusive learning processes. However, research on children's active involvement in concept acquisition implies that pedagogical signals could facilitate exploratory behavior, indicating a complementary relationship. To understand the link between them, we designed an object exploration task for preschool‐aged children featuring between‐subject conditions of pedagogical exploration or pedagogical demonstration. Our findings suggest that if the use of the toy is not demonstrated to children and they are allowed to discover the evidence independently, pedagogical signals do not restrict subsequent exploratory behavior. These results imply that pedagogy and exploration complement each other, with pedagogical signals highlighting the relevant evidence and exploratory behavior enriching knowledge by fostering learning from individual experiences.

## 1 Introduction

The human environment consists of human‐made artifacts whose use is regulated by conventional rules and constrained by the causal structure of the object (Legare and Nielsen 2015). Children acquire tool use mainly via two routes: individual exploration (Gopnik et al. 2004; Ruggeri 2022; Schulz 2012) and learning from knowledgeable others in pedagogical contexts (Csibra and Gergely 2009; Gergely et al. 2007; Koenig and Harris 2005; Shafto et al. 2012).

Studies on the two processes tap into different aspects of learning. Research on exploratory behavior focuses on how children discover the causal structure of the external world. They systematically investigate objects to understand how they work (Schulz 2012), particularly when encountering conflicting or ambiguous evidence (Gweon and Schulz 2008; Perez and Feigenson 2022; Schulz and Bonawitz 2007; Stahl and Feigenson 2015). Young children also consider the context in which they observe the evidence (e.g., either making a toy squeak or labeling it) to infer which other items they can generalize the information to (Gweon et al. 2010; Xu and Denison 2009; Xu and Tenenbaum 2007a, 2007b).

**Summary**

- We investigated whether pedagogy and exploration are mutually exclusive or complementary processes.
- We contrasted how pedagogical signals affect exploratory play during pedagogical exploration and pedagogical demonstration.
- Pedagogical instruction facilitated children to exploit the target evidence but constrained exploration only during pedagogical demonstration.
- The results suggest that pedagogy and exploration are complementary processes.

Exploration offers an opportunity for flexible learning and the possibility to test multiple hypotheses regarding how apparatuses work (Bonawitz et al. 2011; Shneidman et al. 2016; Yu, Landrum, et al. 2018), potentially involving improved memory of the information (Brezack et al. 2021, 2023; Fisher et al. 2013). However, without guidance, exploration may result in limited learning, relying on trial and error. For instance, preschoolers may struggle to uncover information on objects, hindering them from learning about the hidden causal structure of apparatuses (Sobel et al. 2022), or struggle to integrate the discovered evidence into planning further exploration (Meder et al. 2021). Furthermore, some aspects of object knowledge may not be inferred individually, and young children show sensitivity to this: there is evidence that they assume that artifacts are designed for specific purposes (Casler and Kelemen 2007) and that their use is regulated by arbitrary, conventional rules (Schmidt et al. 2012).

Investigations into pedagogy explicate how children learn rapidly generic information or conventional knowledge from their social partners. In a pedagogical context, an adult (“teacher”) communicatively highlights the information to be learned, enabling children to rapidly form inferences about the scope of applicability of that information. According to Bayesian models (Gopnik et al. 2004; Shafto et al. 2012, 2014), children are sensitive to how the information is sampled within a situation. They presume that evidence sampled by adults exhaustively represents the external world and infer, consequently, that all the relevant information has been transmitted to them (Bonawitz et al. 2011; Shneidman et al. 2016; Yu, Landrum, et al. 2018). For instance, Xu and Tenenbaum (2007a) demonstrated that in a word‐learning task, when a teacher matches three identical novel objects with a specific, novel label, their overlapping features, such as color, pattern, shape, or size, provide information about the scope of the word. This, in turn, could lead children to restrictively generalize the object name at a subordinate level, applying it only to items with the exact properties as the labeled ones. However, if children pair the labels with the objects in an “exploratory task”—even if they pick three identical ones—they see the similarities between them as coincidences and thus generalize the label more broadly than in a teaching condition—that is, at a basic level.

Natural pedagogy theory (Csibra and Gergely 2009; Gergely et al. 2007) emphasizes the role of communication in evoking pedagogical contexts. It claims that human communication is specialized to transmit causally opaque, cultural knowledge (such as norms or artifact functions, Diesendruck and Markson 2011) by highlighting significant elements within an action sequence and marking the desired outcome. According to the theory, communicative signals that are ostensive (e.g., eye‐contact, pointing, and infant‐directed speech) indicate the pedagogical intent of the adult and thus the relevance of the communicatively presented information. Indeed, research has demonstrated that children more faithfully imitate sub‐efficient actions (e.g., using the head to turn on a lamp) when an adult emphasizes their actions to the child through ostensive communicative signals, compared to when the same action is observed in a non‐communicative context. In the latter case, children tend to reproduce only the outcome (turning the lamp on) without reproducing the means (using the head) (Király et al. 2013; see also: Egyed et al. 2013; Southgate et al. 2009).

Pedagogy thus enables rapid information transmission, albeit at a price. If the information is flawed, it may compromise the representation of the external world (Gopnik et al. 2004). Results demonstrated that following teaching, children suspend exploring additional information regarding the possible affordances of an object (Bonawitz et al. 2011; Shneidman et al. 2016; Yu, Landrum, et al. 2018) and stick to the taught sub‐efficient action, even if they have observed the more efficient solution in a non‐pedagogical (non‐communicative) context (Hoehl et al. 2014; Marno and Csibra 2015).

Because pedagogy apparently constrains exploration, the two processes are argued to be mutually exclusive (Bonawitz et al. 2011; Gopnik et al. 2004). However, theoretical works on guided play (Toub et al. 2016; Weisberg et al. 2016; Yu, Shafto, et al. 2018) indicate that communicative signals during exploration could serve to highlight the relevance of the discovered information, potentially evoking pedagogical expectations, particularly when adults provide feedback and guidance regarding the playful activities of children. Indeed, research on the active involvement of children in learning could include a teacher providing them with additional information (Fisher et al. 2013), thereby assisting the acquisition of more nuanced knowledge (Ruggeri et al. 2021). Furthermore, it has already been demonstrated that open‐ended “pedagogical” questions transmit relevant knowledge without inhibiting further exploratory learning (Yu, Landrum, et al. 2018). This finding also implies that exploratory behavior is not restricted in all types of pedagogical contexts.

Thus, the link between exploration and pedagogy needs to be clarified. To address this, we designed an experiment inspired by Bonawitz et al. (2011) featuring between‐subjects conditions of pedagogical exploration and pedagogical demonstration, alongside their non‐pedagogical counterparts and a baseline condition. The experimental conditions were designed by varying two parameters. First, children either independently explored the apparatus until they found an affordance or observed the experimenter demonstrate one. Second, the context was either pedagogical, where the adult communicated about the target affordance (whether explored or demonstrated), or non‐pedagogical, where the communication was unrelated to the apparatus. In the baseline condition, children played with the toy freely, without any intervention. Importantly, the pedagogical demonstration, non‐pedagogical demonstration, and baseline conditions were replications of the original study by Bonawitz et al. (2011). We included these conditions in our study for two main reasons: first, to enable a comparison between our results and those of the original research, and second, to build a more comprehensive body of evidence regarding the effect of our intervention.

Note that we examined how pedagogical signals influence further information acquisition in settings where evidence is either discovered by children or demonstrated by an adult. In line with the theory of natural pedagogy (Csibra and Gergely 2009; Gergely et al. 2007), we define pedagogy as a context in which an adult ostensively communicates the relevance and generalizability of the presented evidence. Importantly, we view pedagogical contexts not as episodes in which children are passive recipients of information from adults, but rather as situations in which the relevance of information is emphasized through social interaction. In the present study, the pedagogical context was established through a verbal utterance that was both contingent upon and informative about the activation of the target affordance.

Following the original paradigm (Bonawitz et al. 2011), object exploration can be characterized by how long children play with the artifact, how much of that time they spend exploiting the target affordance (indicating their assumptions about the relevance of the information and illustrating the effectiveness of the instruction in transmitting knowledge), and the variety of ways they interact with the object (exploratory behavior). This variety is measured by the number of unique action‐kinds and the number of non‐target affordances discovered during play, indicating whether children consider the possibility that the object has additional causal properties or alternative uses.

Regarding the critical pedagogical exploration condition, the prediction based on previous studies could be that pedagogy highlights the relevance of the discovered evidence and thus inhibits further exploration. This would be reflected in a relatively long engagement with the target evidence, a small number of unique action‐kinds, and a limited number of discovered non‐target affordances in the pedagogical exploration condition. In contrast, a complementary approach‐based assumption would be that pedagogy highlights the relevance of the evidence and, at the same time, promotes exploratory behavior by reinforcing not just the action that produces an effect on the toy but all the distinct actions children exhibited before the intervention. This would be indicated by a relatively long engagement with the target affordance, a high number of unique action‐kinds, and a high number of discovered non‐target affordances in the pedagogical exploration condition.

Based on earlier findings (Bonawitz et al. 2011; Shneidman et al. 2016; Yu, Landrum, et al. 2018), we anticipated that exploratory behavior would be limited in the pedagogical demonstration condition—because the instruction prompts children to focus on the presented evidence—indicated by a relatively long engagement with the target affordance and low variety in the exploratory actions produced during play. Importantly, both the complementary approach‐based hypothesis and the mutual exclusivity approach‐based hypothesis predict limited exploratory learning, as the broader context itself does not promote the deeper discovery of the toy.

We expected that children's exploratory play in the non‐pedagogical conditions would not be focused on exploiting the target information. As a result, we predicted a relatively short playtime with the target affordance, a high number of unique action‐kinds, and a high number of non‐target affordances discovered in the non‐pedagogical exploration, non‐pedagogical demonstration, and baseline conditions.

Our prediction about how playtime would differ across conditions was less clear. On the one hand, research has found that children explore a toy for a longer period if they assume there is more to discover (Bonawitz et al. 2011, Experiment 1). Therefore, a relatively short period of overall playtime in the pedagogical exploration and pedagogical demonstration conditions could imply limited exploratory behavior. On the other hand, studies have also demonstrated that playtime can be independent of children's inferences about the toy: children may spend the same amount of time playing with the toy whether they engage with only one affordance or explore multiple affordances (Bonawitz et al. 2011, Experiment 2). This indicates that playtime is not a foolproof measure of exploratory learning.

## 2 Methods

Children were tested individually in a quiet room either in our laboratory or in local kindergartens. The study was carried out with the approval of the Research Ethics Committee of the Faculty of Education and Psychology of Eötvös Loránd University. Caregivers gave written informed consent.

### 2.1 Participants

Following previous research (Yu, Landrum, et al. 2018) and ensuring that all affordances are demonstrated an equal number of times in the demonstration conditions, the planned sample size for the experimental conditions was 128 participants (32 per condition). We tested an additional 20 children in the baseline condition, in light of previous research with smaller sample sizes for the baseline group (e.g., Altınok et al. 2024; Hoehl et al. 2014).

Children were randomly assigned to one of five conditions: (1) pedagogical demonstration (PedDem), (2) pedagogical exploration (PedExp), (3) non‐pedagogical demonstration (NpedDem), (4) non‐pedagogical exploration (NpedExp), and (5) a baseline. Age and gender were counterbalanced.

The final sample included 148 preschoolers (mean age: 58.77 months; range: 48–72 months; 76 boys) (data collection occurred in Hungary, where data on race/ethnicity cannot legally be collected). An additional 34 children were tested but replaced due to passivity (*n*_PedExp&NpedExp_ = 10; *n*_PedDem_ = 2; *n*_NpedDem_ = 3; *n*_Baseline_ = 5), experimenter error (*n*_PedDem_ = 1; *n*_PedExp_ = 1; *n*_NpedDem_ = 1; *n*_Baseline_ = 5), camera failure (*n*_Baseline_ = 1), age beyond range (*n*_Baseline_ = 4), and testing interference (*n*_PedDem_ = 1). Importantly, the “passive” behaviors in both the pedagogical exploration and non‐pedagogical exploration conditions occurred during the exploration phase of those conditions: we excluded and replaced those children who were either reluctant or unable to explore the toy and discover one of its affordances.

### 2.2 Materials

**FIGURE 1:**
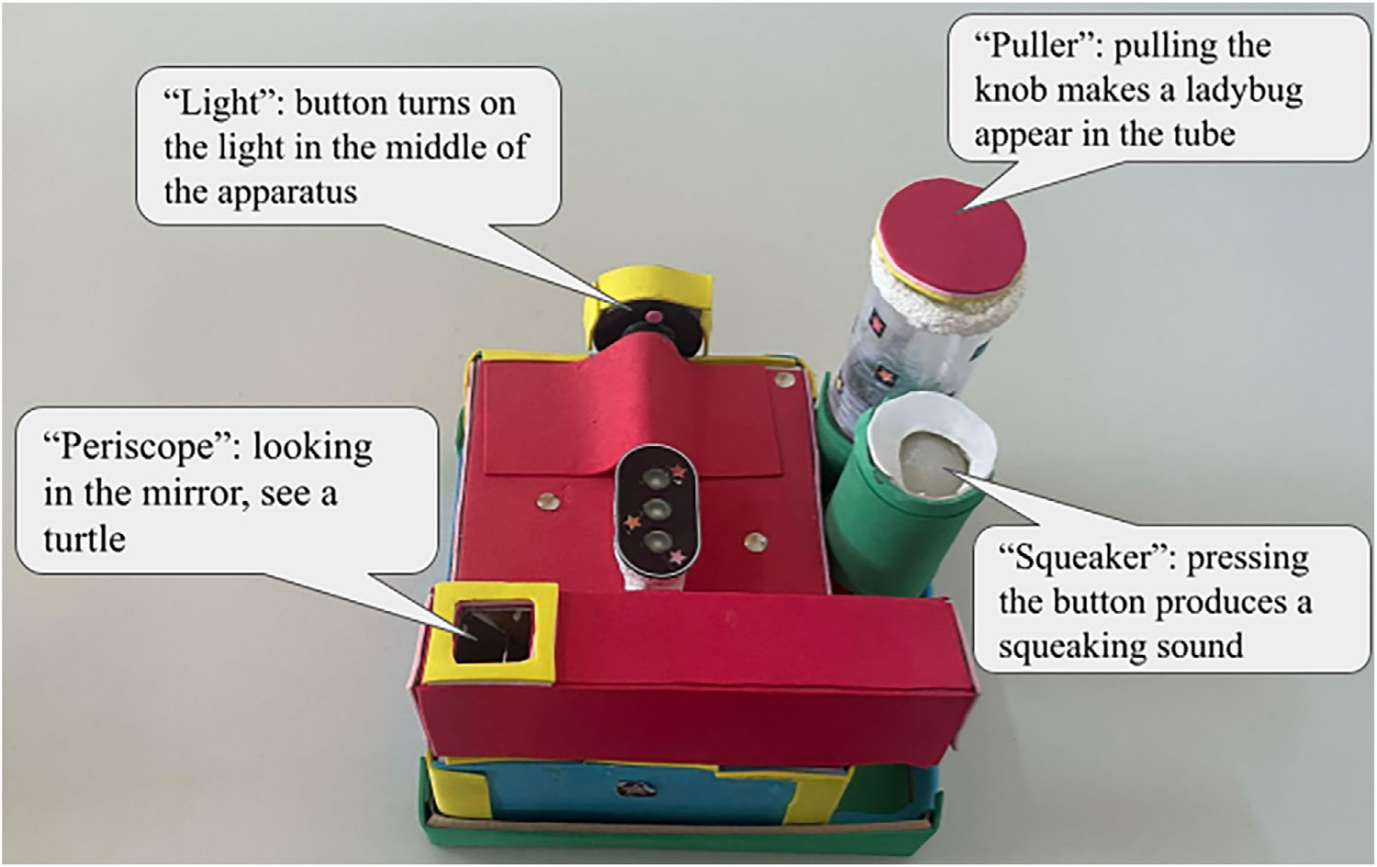
The apparatus used in the experiment.

A novel toy (Figure 1) was created, measuring about 20 × 20 × 8 cm. It featured four affordances: (1) pressing a button produced a squeaking sound, (2) pressing a switch illuminated lights in the center, (3) pulling a transparent rope caused a ladybug to ascend in a tube, and (4) peering through a periscope revealed a turtle.

### 2.3 Procedure

The procedure was based on the research of Bonawitz et al. (Experiment 2, 2011). The child was seated at a table. The experimenter (E) sat on their right side, perpendicular to them. The apparatus was hidden beneath the table, covered by a blanket. First, the experimenter (E) placed the object on the table, out of reach of the child. In the pedagogical demonstration condition, E said, “Look at this toy! Watch this!” E then demonstrated one of the affordances and said, “This is how this toy can be played with!” The non‐pedagogical demonstration condition was identical to the pedagogical demonstration condition, except that immediately after the demonstration, E interrupted themselves and said, “I just realized I forgot to finish something. I have to go there and take care of it right now!” Importantly, E's comment was not informative about the demonstrated affordance. The demonstration of the affordances was counterbalanced.

In the pedagogical exploration and the non‐pedagogical exploration conditions, E said after putting the apparatus on the table, “Look at this toy! Play with it!” and placed it in front of the child and let them explore it on their own. If the child asked for help, E said neutrally, “Play with it! You are doing great!” When the child discovered the first affordance, E interrupted the play by taking the object away from the child. In the pedagogical exploration condition, E then said, “Super! This is how this toy can be played with!” In the non‐pedagogical exploration condition, E simply said, “Super.” In both conditions, children were allowed to use the discovered affordance only once, because E interrupted their play immediately after they successfully discovered the first affordance. In the baseline condition, E put the apparatus on the table and then said, “Look at this toy!” and looked at it with the child for 3 s before leaving them alone.

In all conditions, E then said, “I have some things to do, so I have to go sit over there and work a bit on my things. You stay here and play with the toy. Let me know when you are done!” and left the child alone at the table to play with the toy.

During the response phase, E sat in a corner of the room, pretending to work. E did not respond to the child's comments or interact with them during this phase. If the child said they finished playing or had been playing with the toy for more than 10 minutes, E terminated the experiment. If the child stopped touching the artifact for more than 5 consecutive seconds, E asked them, “Are you done?” E terminated the testing if the child answered yes. Otherwise, E let the child continue to play. E then finished the experiment if the child stopped interacting with the toy for a second time for more than 5 seconds.

### 2.4 Coding

All sessions were videotaped and coded by an experimenter. A second independent observer—who was blind to the hypotheses of the study—coded all sessions for reliability. The coding followed the protocol of the Bonawitz et al. (2011) study.

We coded time to explore the target affordance (in the baseline, pedagogical exploration, and non‐pedagogical exploration conditions), total playtime (only in the response phase), time spent with the target affordance (initially discovered or demonstrated), number of unique action‐kinds performed on the apparatus (including successful or unsuccessful non‐target activations), and number of discovered non‐target affordances (including only successful non‐target activations). Reliability was very good (elapsed time until discovering the target: *R^2^* = 0.99, total playtime: *R^2^* = 0.99; time playing with target affordance: *R^2^* = 0.91; unique action‐kinds: Cohen's weighted *κ* = 0.84; discovered affordances: Cohen's weighted *κ* = 0.98).

## 3 Results

Statistics were computed using R 4.3.2 (R Core Team 2023) with lme4 (Bates et al. 2014) and emmeans (Lenth 2023) packages. All data and detailed results have been made publicly available at https://doi.org/10.17605/OSF.IO/X5FNY.

Linear contrasts and mixed‐effects linear models were used to investigate the outcomes for the dependent variables: total playtime, proportion of time playing with the target affordance, number of unique action‐kinds, and number of discovered (non‐target) affordances. The proportion of time spent playing with the target affordance was calculated by dividing the time spent on the target affordance by the total playtime, excluding the time taken to discover the first affordance in the baseline condition.

### 3.1 Contrasting the Experimental Conditions

Following the analytical approach of the original study (Bonawitz et al. 2011), we tested our two hypotheses about the relationship between exploratory learning and pedagogy (complementary approach‐based and mutual exclusivity‐based) using linear contrasts with weights adjusted to reflect the two competing predictions. The pedagogical demonstration contrast (CPedDem; weights: −3, 1, 1, 1) investigated whether the pedagogical demonstration condition differed from the pedagogical exploration, non‐pedagogical demonstration, and non‐pedagogical exploration conditions. The pedagogical contrast (CPed; weights: −1, −1, 1, 1) examined whether the two pedagogical conditions differed from the two non‐pedagogical conditions. As we applied two contrasts to investigate our research question, the significance level was adjusted using Bonferroni correction.

Based on the mutual exclusivity approach, the pedagogical contrast should be significant across all measurements, indicating that pedagogical instruction limits exploratory behavior in all learning contexts by prompting children to focus solely on exploiting the target evidence. Conversely, based on the complementary approach, the pedagogical contrast should be significant only in the case of the proportion of time spent on playing with the target affordance, which would provide evidence that the “pedagogical instruction” effectively transmitted knowledge. In all the other measurements, the pedagogical demonstration contrast should be significant, illustrating that pedagogical instructions do not restrict exploratory behavior across all pedagogical contexts. Note that we had less clear expectations on how playtime should differ between the experimental conditions.

**FIGURE 2:**
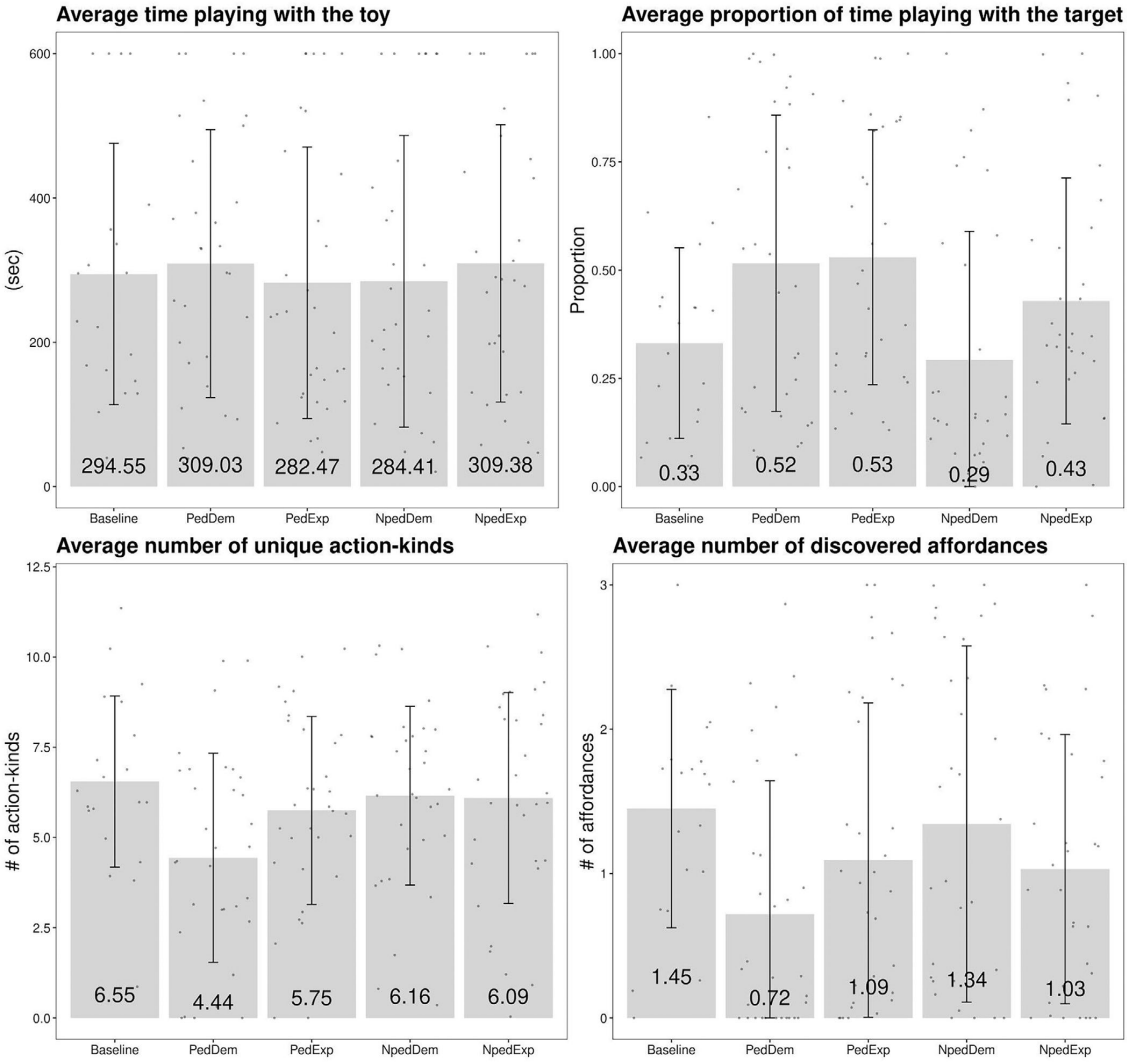
Results by condition. Bar plots depict the average responses by condition. Scatter plots depict individual participants’ jittered performance.

In the case of total playtime (*M_PedDem_* = 309.03, *M_PedExp_* = 282.47, *M_NpedDem_* = 284.41, *M_NpedExp_* = 309.38), none of the contrasts were significant (CPedDem: *F*(1,124) = 0.187, *p* = 0.666; CPed: *F*(1,124) = 0.001, *p* = 0.973). In the case of the proportion of time spent on playing with the target affordance (*M_PedDem_* = 0.52, *M_PedExp_* = 0.53, *M_NpedDem_* = 0.29, *M_NpedExp_* = 0.43), the pedagogical contrast was significant (CPedDem: *F*(1,124) = 2.508, *p* = 0.116; CPed: *F*(1,124) = 9.021, *p* = 0.003, *η^2^* = 0.066). In the case of the number of unique action‐kinds (*M_PedDem_* = 4.44, *M_PedExp_* = 5.75, *M_NpedDem_* = 6.16, *M_NpedExp_* = 6.09), the pedagogical demonstration contrast was significant (CPedDem: *F*(1,124) = 7.856, *p* = 0.006, *η^2^* = 0.059; CPed: *F*(1,124) = 4.563, *p* = 0.035). None of the contrasts were significant in the case of the number of discovered affordances (*M_PedDem_* = 0.72, *M_PedExp_* = 1.09, *M_NpedDem_* = 1.34, *M_NpedExp_* = 1.03; CPedDem: *F*(1,124) = 4.146, *p* = 0.044; CPed: *F*(1,124) = 2.285, *p* = 0.113). See Figure 2 for details.

The results of the contrasts support the prediction derived from the complementary approach‐based hypothesis. The two pedagogical conditions differed from the non‐pedagogical conditions only when analyzing the proportion of time children spent playing with the target affordance. For the number of unique action‐kinds, the pedagogical demonstration condition differed significantly from the other conditions. However, for total playtime and, unexpectedly, the number of discovered affordances, none of the contrasts were significant.

Note that in the case of the number of discovered affordances, the insignificant difference may be partially attributed to our conservative measurement, which did not account for instances where children unsuccessfully attempted to activate non‐target affordances (e.g., they pushed the “squeaker” but it did not make a sound, or they only partially pulled the knob of the “puller” and did not see the ladybug on the ball). Considering that, in this paradigm, non‐target affordance discovery results from trial and error information search enabled by unrestricted exploratory play, and when analyzing the number of unique action‐kinds, the pedagogical demonstration contrast was significant (indicating that exploration is limited only in the pedagogical demonstration condition), we could have expected the pedagogical demonstration contrast to also be significant for the number of discovered affordances, similar to the findings in the original Bonawitz et al. (2011) study. Additionally, note that, unlike our study, Shneidman et al. (2016), a direct replication of the original study, coded unsuccessful non‐target activations as affordance discoveries.

### 3.2 Comparing the Experimental Conditions to the Baseline Condition

We examined how the different conditions influenced the way children learned about the apparatus. We reasoned that the baseline condition provided the “purest” setting for exploratory learning, as it involved no interventions. Therefore, to fully capture the constraints and potential enrichments (i.e., the possibility to recognize and deeply explore the relevant evidence) provided by pedagogical signals during learning, we fitted regression models with the baseline condition set as the reference value for comparison.

We fit mixed‐effects linear models with maximum likelihood estimation for our dependent variables, adding random intercepts for each location of data collection. The analysis plan for all model comparisons employed a stepwise approach, where fixed effects (age first, then gender, and condition) were added sequentially to the model. At each step, the more complex model was compared to the best‐fitting simpler model below it in the hierarchy, starting with the null model. We report marginal *R^2^* as a measure of effect size.

Initially, as an exploratory analysis, we tested the effect of age and gender. The model with age significantly outperformed the null model for several dependent measures but not for total playtime. Therefore, age was included as a fixed effect in all models (including the “null” models) except for total playtime.

For each analysis, we compared a model with a fixed effect for the condition to a “null model.” The model with the condition did not perform better than the null model for the total playtime (*χ2*(4) = 0.879, *p* = 0.928) and outperformed it for the proportion of time playing with the target affordance (*χ2*(4) = 15.844, *p* = 0.003, *R^2^* = 0.133), the number of unique action‐kinds (*χ2*(4) = 13.790, *p* = 0.008, *R^2^* = 0.128), and the number of discovered affordances (*χ2*(4) = 10.483, *p* = 0.033, *R^2^* = 0.124). See Figure 2 for details.

Compared to the baseline, children spent significantly more time playing with the target affordance when receiving pedagogical instruction (age: *β* = −0.125, SD = 0.052, 95% CI [−0.23, −0.02], *p* = 0.019; PedDem: *β* = 0.186, SD = 0.082, 95% CI [0.02, 0.35], *p* = 0.024; PedExp: *β* = 0.181, SD = 0.082, 95% CI [0.02, 0.34], *p* = 0.029; NpedDem: *β* = −0.051, SD = 0.082, 95% CI [−0.21, 0.11], *p* = 0.532; NpedExp: *β* = 0.087, SD = 0.082, 95% CI [−0.07, 0.25], *p* = 0.286).

Additionally, children produced significantly fewer unique action‐kinds (age: *β* = 1.341, SD = 0.493, 95% CI [0.37, 2.32], *p* = 0.008; PedDem: *β* = −2.374, *SD* = 0.762, 95% CI [−3.88, −0.87], *p* = 0.002) and discovered significantly fewer affordances (age: *β* = 0.592, SD = 0.186, 95% CI [0.22, 0.96], *p* = 0.002; PedDem: *β* = −0.757, SD = 0.288, 95% CI [−1.22, −0.19], *p* = 0.010) in the pedagogical demonstration condition compared to the baseline condition.

The other conditions did not differ significantly from the baseline in the number of unique action‐kinds (PedExp: *β* = −0.799, SD = 0.761, 95% CI [−2.30, 0.71], *p* = 0.296; NpedDem: *β* = −0.481, SD = 0.767, 95% CI [−2.00, 1.04], *p* = 0.532; NpedExp: *β* = −0.533, SD = 0.761, 95% CI [−2.04, 0.97], *p* = 0.485) and the number of discovered affordances (PedExp: *β* = −0.276, SD = 0.287, 95% CI [−0.84, 0.29], *p* = 0.338; NpedDem: *β* = −0.086, *SD* = 0.290, 95% CI [−0.66, 0.49], *p* = 0.768; NpedExp: *β* = −0.371, SD = 0.287, 95% CI [−0.94, 0.20], *p* = 0.198).

Thus, compared to the baseline, children spent more time exploiting the target following pedagogical instruction. Exploratory behavior was limited only in the pedagogical demonstration condition. Older children engaged less with the target and explored the toy more thoroughly.

### 3.3 Exploratory Behavior in the Pedagogical Exploration, Non‐Pedagogical Exploration, and Baseline Conditions

We further examined whether pedagogical feedback affected exploration by reinforcing actions performed before target discovery. In the pedagogical exploration, non‐pedagogical exploration, and baseline conditions, unique actions were categorized as discontinued (the behavior was produced before discovering the target information and stopped being exhibited afterward), post‐discovery (the behavior was initiated only after discovering the target information), or continued (the behavior was produced both before and after discovering the target information). We then calculated a novelty preference score for each child by summing the proportion of post‐discovery actions and the proportion of discontinued actions while subtracting the proportion of continued actions. A score of 1 would indicate a tendency to exhibit predominantly post‐discovery behaviors, and a score of −1 would suggest a preference for reverting to pre‐discovery actions during the response phase. (Note that we could not calculate the novelty score for one of the participants in the pedagogical exploration condition due to a recording error during the exploratory phase.) The models with fixed effects for gender, age, or condition (*χ^2^* (2) = 0.492, *p* = 0.782) did not perform better than the null model.

**FIGURE 3:**
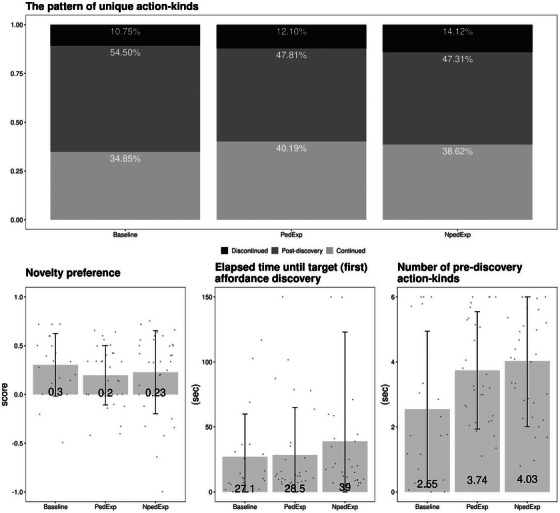
Patterns of exploratory behavior. Stacked plots depict the distribution of the proportions of action categories in the baseline, pedagogical exploration, and non‐pedagogical exploration conditions. Bar plots depict the average responses by condition. Scatter plots depict individual participants’ jittered performance.

As an exploratory analysis, we investigated how children discovered the target affordance by comparing the number of unique pre‐discovery actions and elapsed time until discovering the first (target) affordance across the pedagogical exploration, non‐pedagogical exploration, and baseline conditions. None of the models with fixed effects for age, gender, or conditions (pre‐discovery actions: *χ^2^* (2) = 3.983, *p* = 0.137; time until first affordance discovery: *χ^2^* (2) = 0.790, *p* = 0.674) showed superior performance over the null models. Consequently, there was no difference between the conditions in how children discovered the first affordance or planned and initiated their exploratory behavior. This implies that the pedagogical instructions did not facilitate exploratory behavior per se by reinforcing the specific behaviors performed by children while searching for the first affordance. See Figure 3 for details.

### 3.4 The Effect of Instruction Type, Information Delivery, and Their Interaction

Additionally, to investigate how the mode of information delivery and the instruction influenced the behaviors observed in each experimental condition, we compared a model with Information delivery (demonstration or exploration), a model with Instruction type (pedagogical or non‐pedagogical), and a model with their interaction. Each more complex model was always compared to the best‐fitting simpler model below it in the hierarchy, starting with a comparison to the null model.

For the total playtime, neither model outperformed the null model. The model with the instruction type was the best fit for explaining the proportion of time playing with the target (*χ^2^* (1) = 9.488, *p* = 0.002, *R^2^* = 0.127) and the number of unique action‐kinds (*χ^2^* (1) = 5.313, *p* = 0.021, *R^2^* = 0.076): children played significantly longer with the target affordance (Ped vs. Nped: *β* = −0.166, SD = 0.053, *p* = 0.002; *M_Ped_* = 0.52 SD = 0.32; *M_Nped_* = 0.36 SD = 0.30) and produced fewer number of unique action‐kinds (Ped vs. Nped: *β* = 1.079, SD = 0.462, *p* = 0.021; *M_Ped_* = 5.09 SD = 2.81; *M_Nped_* = 6.12 SD = 2.69) following pedagogical instructions compared to when receiving non‐pedagogical ones.

For explaining the number of discovered affordances, the model with the interaction was the best fit (*χ^2^* (3) = 7.944, *p* = 0.047, *R^2^* = 0.115; Exp vs. Dem: *β* = 0.479, SD = 0.248, *p* = 0.056; Ped vs. Nped: *β* = 0.691, SD = 0.247, *p* = 0.006; Interaction: *β* = −0.792, SD = 0.350, *p* = 0.025). Post hoc comparisons revealed that following pedagogical instruction, children discovered significantly fewer affordances when the adult demonstrated the target evidence (*t*(123) = −2.743, *p* = 0.007). The success of the discovery was not altered when children found it (*t*(123) = 0.401, *p* = 0.689).

## 4 Discussion

We investigated whether exploration and pedagogy are mutually exclusive or complementary processes by inviting preschoolers to play with a novel toy with four affordances. The results of the linear contrasts suggest that the two processes are complementary: pedagogical signals promoted deeper exploration of the target affordance but constrained exploratory behavior only when the pedagogical instruction was preceded by the demonstration of the target evidence. Moreover, when comparing the experimental conditions to the baseline, we also found that, following pedagogical instructions, children spent significant time playing with the target affordance. However, pedagogical instruction constrained the initiation of unique actions and affordance discovery only when pedagogical instructions were presented together with the demonstration of the target information. These results further support the idea that exploration and pedagogy work together to enhance learning.

The results did not support the prediction that pedagogical instructions might promote the further exploration of the actions performed before discovering the target affordance: there was no difference between the pedagogical exploration, non‐pedagogical exploration, and baseline conditions in terms of the novelty preference score. Children might have inferred from the pedagogical feedback that they should continue exploring the toy, which involves exploiting the target evidence and discovering new ones.

Additionally, older children played less with the target and explored the toy more intensively and effectively. Previous research has demonstrated that they also incorporate novel evidence more flexibly (Altınok et al. 2020). Furthermore, Bass and Bonawitz (2024)—by analyzing data from past experiments on exploratory learning—also demonstrated a similar effect of age on the variability of exploratory behavior. Altogether, these findings could imply that children become more efficient and less reliant on the help of adults in planning their learning as they grow older.

This study also extends the literature on natural pedagogy (Csibra and Gergely 2009; Gergely et al. 2007) by disentangling the role of communication and demonstration in creating pedagogical contexts. On the one hand, our study confirmed with additional data, that pedagogical signals mark the relevant evidence in the learning context. Such cues might lead children to faithfully reproduce the demonstrated action—as they assume it is the normative behavior (Schmidt et al. 2012) or the way the artifact is designed to be used (Casler and Kelemen 2007)—and consequently, this may constrain their exploratory behavior (Bonawitz et al. 2011; Shneidman et al. 2016; Yu, Landrum, et al. 2018; see further discussion on this question in Altınok et al. 2024). At the same time, this study also demonstrates that pedagogy does not necessarily limit discovering or recognizing causal information hidden within a device. Therefore, if the context allows (because it is playful) or forces (because it involves challenges) children to drop the previously transmitted strategy, children could invent novel solutions or modify the learned ones.

Importantly, in this study, instructed exploratory contexts included pedagogical instruction and omitted demonstration. This design aimed to discern how communication alone evokes pedagogical expectation while ensuring children witness the effect of the target evidence equally in all conditions. Consequently, our results demonstrate that pedagogy manifests itself in ostensive‐communicative signals and not exclusively in didactic information transmission. This finding aligns with anthropological studies reporting that, in rural societies, adults help children master hunting by determining what toys they play with and giving feedback about their behavior (MacDonald 2007; Watanabe 1975). However, further research is required to comprehensively understand the inferences children draw during guided exploration, including assessing generalization or resistance to counterevidence.

It is noteworthy that while this study primarily focused on examining the relationship between learning from pedagogical signals and exploratory behavior, the findings of this experiment may also inform educational practices. The pedagogical exploration condition and the pedagogical demonstration condition may resemble guided play in a “natural” educational context and didactic teaching, respectively. Guided play (Toub et al. 2016; Weisberg et al. 2016; Yu, Landrum, et al. 2018) has been argued to be a learning approach that combines the benefits of both independent and directed learning. During guided play, the child exercises autonomy over their information search and learning, while the teacher's role is to highlight relevant information and correct inaccuracies through feedback. Didactic teaching, on the other hand, employs a more passive transmission of information, which may limit children's further exploration (Bonawitz et al. 2011; Shneidman et al. 2016; Yu, Landrum, et al. 2018).

The results of this study suggest that children are highly receptive to pedagogical signals. However, when exploratory behavior is also promoted, as in guided play (e.g., using pedagogical questions or instructions that facilitate exploring the evidence; Fisher et al. 2013; Yu, Landrum, et al. 2018), pedagogical signals, although prompting children to exploit the highlighted information in depth, do not inhibit the possibility of exploring and examining alternative evidence. This implies that guided play is as effective at transmitting information as didactic teaching, while also promoting children's autonomy in testing alternative possibilities. This may lead to more joyful learning in formal education and improved academic performance.

Note that when we contrasted the mode of information delivery and the type of instruction to analyze the experimental conditions, we found that pedagogical instruction generally influenced exploratory behavior during both demonstrative and exploratory settings: children played longer with the target and explored less following pedagogical instructions. While we consider this result as suggestive of children being sensitive to the nature of the instruction provided by their social partners, we argue that more conclusive evidence regarding the impact of pedagogical instruction on exploratory behavior can be obtained when the individual conditions are contrasted directly with each other.

Moreover, despite the four experimental conditions being designed by varying the mode of information delivery and the type of instruction, the context of the different conditions presumably did not differ solely along these factors. For instance, in the non‐pedagogical exploration condition, the intervention could signal that children found the relevant information, fulfilling the goal set by the adult. In contrast, in the non‐pedagogical demonstration condition, children possibly did not interpret the experimenter's self‐interruption as meaningful regarding the target information. This possibility limits the interpretation of the contrasts.

Altogether, our results suggest that pedagogy and exploration complement each other by promoting information acquisition in different ways: pedagogical cues highlight what information is relevant within a context, and exploratory behavior fosters an understanding of the causal structure, thereby contributing to a more elaborate and enriched knowledge of objects or problems (Brezack et al. 2021). Arguably, pedagogy should not necessarily interfere with exploratory behavior but rather emphasize which evidence is worth exploring in greater depth. This proposal is consistent with the absence of difference in playtime across conditions. Given that play can be driven by various factors, such as discovering novel information or exploiting known ones (Chu and Schulz 2023), prolonged play with the target could offer insights into its causal structure. To conclude, we provided evidence that pedagogy and exploratory behavior work in tandem to support learning about objects.

## Ethics Statement

All the experiments presented in the study were approved by the ethical committee of the University (Ethical Committee of the Eötvös Loránd University, Budapest, approval No. 2022/420‐2)

## Consent

Permission to reproduce material from other sources: non‐applicable.

## Conflicts of Interest

There authors declare no conflicts of interests.

## Data Availability

The data that support the findings of this study are openly available in OSF at: https://doi.org/10.17605/OSF.IO/X5FNY
